# IgA nephropathy and kidney transplantation according to the Oxford classification

**DOI:** 10.1590/2175-8239-JBN-2022-0051en

**Published:** 2023-01-09

**Authors:** André de Sá Vasconcelos, Marilda Mazzali, Marcos Vinicius de Sousa

**Affiliations:** 1 Universidade Estadual de Campinas, Faculdade de Ciências Médicas, Departamento de Clínica Médica, Divisão de Nefrologia, Unidade de Transplante Renal, Laboratório de Investigação em Transplante, Campinas, SP, Brasil.

**Keywords:** Kidney Transplantation, Glomerulonephritis, Immunoglobulin A, Histologia, Recurrence, Renal Insufficiency, Chronic, Transplante de Rim, Glomerulonefrite, Imunoglobulina A, Histology, Recidiva, Insuficiência Renal Crônica

## Abstract

**Introduction:**

IgA nephropathy (IgAN) is the most common glomerular disease globally, and its susceptibility and the risk for the development of end-stage kidney disease are related to genetic and environmental factors. IgAN recurrence after kidney transplantation is relatively common, impacting graft function and survival. This study evaluated the risk factors and the clinical, laboratory, and histological characteristics of post-transplant IgAN recurrence based on the Oxford classification.

**Material and methods:**

Retrospective single-center cohort study including kidney transplant recipients with biopsy-proven pre-transplantation IgAN, with analysis of risk factors and clinical, laboratory, and histological characteristics of the IgAN recurrence cases.

**Results:**

53 patients fulfilled the inclusion criteria and were included in the study. The majority was male, white, eutrophic, with a mean age of 27 ± 9 years at IgAN diagnosis. Systemic arterial hypertension and proteinuria were frequent in the pretransplant period. Four recipients (7.5%) presented IgAN recurrence in a period of 6 to 122 months post-transplant. According to the Oxford classification, they had high scores of mesangial hypercellularity and segmental glomerulosclerosis in the native kidney biopsies and there was mesangial hypercellularity in all analyzed graft biopsies. None of these patients had received induction immunosuppression and all of them presented graft failure in the follow-up.

**Conclusions:**

In this series, there was a high prevalence of mesangial hypercellularity and segmental glomerulosclerosis on native kidney biopsies, and mesangial hypercellularity occurred in all IgAN recurrence graft biopsies. Despite the lower incidence of recurrence of IgAN post-transplant compared to previous reports, progression to graft loss was of 100%.

## Resumo

**Introdução:**

Nefropatia por IgA (NIgA) é a doença glomerular mais comum mundialmente. Sua suscetibilidade e risco para desenvolvimento de doença renal em fase terminal estão relacionados a fatores genéticos e ambientais. A recidiva de NIgA pós-transplante é relativamente comum, impactando na função e sobrevida do enxerto. Este estudo avaliou fatores de risco e características clínicas, laboratoriais e histológicas da recidiva de NIgA pós-transplante, com base na classificação de Oxford.

**Material e métodos:**

Estudo de coorte retrospectivo de centro único, incluindo receptores de transplante renal com NIgA pré-transplante comprovada por biópsia, com análise dos fatores de risco e características clínicas, laboratoriais e histológicas dos casos de recidiva de NIgA.

**Resultados:**

53 pacientes preencheram critérios de inclusão e foram incluídos no estudo. A maioria era homem, branco, eutrófico, com idade média de 27 ± 9 anos no diagnóstico de NIgA. Hipertensão arterial sistêmica e proteinúria foram frequentes no período pré-transplante. Quatro receptores (7,5%) apresentaram recidiva de NIgA entre 6-122 meses pós-transplante. Segundo a classificação de Oxford, eles apresentaram altos escores de hipercelularidade mesangial e glomeruloesclerose segmentar nas biópsias de rins nativos. Houve hipercelularidade mesangial em todas as biópsias de enxerto analisadas. Nenhum destes pacientes recebeu imunossupressão de indução. Todos apresentaram falência do enxerto no acompanhamento.

**Conclusões:**

Nesta série, houve alta prevalência de hipercelularidade mesangial e glomeruloesclerose segmentar em biópsias de rins nativos, e hipercelularidade mesangial ocorreu em todas as biópsias do enxerto de recidiva da NIgA. Apesar da menor incidência de recidiva de NIgA pós-transplante comparada a relatos anteriores, a progressão para perda do enxerto foi de 100%.

## Introduction

IgA nephropathy (IgAN) is the most common glomerular disease globally, with prevalence varying geographically according to kidney biopsy indications^1^. The susceptibility to IgAN and chronic kidney disease (CKD) risk are related to genetic and environmental factors^1^. Coexisting risk factors, such as hypertension, smoking, and obesity, contribute to microvascular injury and disease progression, with CKD rates reaching 20% after 20 years of follow-up^1,2^. IgAN results from circulating and glomerular complexes comprised of galactose-deficient IgA1 directed against the hinge region O-glycans, leading to glomerular inflammation and mesangial proliferation^1^. The clinical presentation of IgAN ranges from microscopic hematuria during respiratory or gastrointestinal tract infections in children and young adults to impaired kidney function in older adults, with proteinuria and high blood pressure^2^. IgAN recurrence after kidney transplantation is relatively common, with a 15-year cumulative incidence of 15%^3^. Several risk factors for recurrence of IgAN post-transplant have been described, including younger age, recipients of zero-HLA mismatched living donors, steroid-avoidance or early steroid withdrawal, absence of induction immunosuppressive therapy, HLA allelic subtypes, crescentic and rapidly progressive native IgAN, and shorter total ischemia time^3^.

The hallmark of IgAN diagnosis is predominant IgA deposits in the mesangium, isolated or associated with IgG and/or IgM, C3, properdin, C4 or C4d, mannose-binding lectin, and terminal complement complex (C5b-C9), while C1q is usually absent^2^. Electron microscopy usually shows electron-dense deposits in the mesangial and paramesangial areas and occasionally in the sub-endothelial and sub-epithelial regions^4^. IgAN commonly presents with mesangial expansion and hypercellularity in light microscopy, but focal glomerular necrosis, segmental sclerosis, and crescents in Bowman's space may also be detected^4^. The Oxford IgAN classification defines four histopathological features with prognostic value: mesangial hypercellularity (M), endocapillary hypercellularity (E), segmental glomerulosclerosis (S), and tubular atrophy/interstitial fibrosis (T), commonly referred to as the MEST score^5^. A recent update added cellular and fibrocellular crescents (C) to previous criteria, resulting in the MEST-C score^6^.

The present study evaluated risk factors and clinical, laboratory, and histological characteristics of post-transplant IgAN recurrence based on the Oxford IgAN classification.

## Material and Methods

This was a retrospective single-center cohort study including kidney transplant recipients with biopsy-proven pre-transplantation IgAN. Patients younger than 18 years and those without histologic confirmation of IgAN were excluded. Clinical, laboratory and histological data were retrospectively collected from medical records and renal transplant program databases at the time of transplantation and during the post-transplant follow-up. Data were transcribed and organized into a Microsoft™ Excel worksheet. The study was approved by the local ethics committee.

Primary outcomes were risk factors for IgAN recurrence and clinical, laboratory, and histological characteristics of these cases. The secondary endpoint comprised analysis of the histologic features of cases with an available description of Oxford parameters in native kidney biopsies according to IgAN recurrence after transplantation.

Induction immunosuppressive therapy consisted of basiliximab (monoclonal anti-IL-2 receptor antibody) in recipients with panel-reactive antibody (PRA) lower than 50%, without anti-HLA donor-specific antibodies (DSA) receiving a kidney from a standard deceased donor. In cases of expanded criteria donors, non-identical HLA living donors, PRA > 50%, or presence of DSA, induction therapy was with IV anti-thymocyte globulin, 3-7 mg/kg, dose-adjusted by lymphocyte count. All recipients received 500 mg methylprednisolone IV at the time of transplantation and steroid therapy during follow-up. Maintenance immunosuppression consisted of a calcineurin inhibitor (tacrolimus or cyclosporine) and an antiproliferative drug (sodium mycophenolate or azathioprine).

The post-transplant allograft biopsies were performed if serum creatinine increased more than 20% from baseline or if new-onset proteinuria occurred. Two experienced renal pathologists performed the histologic analysis. The rejection cases were diagnosed and categorized based on histological findings and blood DSA detection according to the 2013 Banff classification, revised in 2015^7^. The IgAN recurrence was diagnosed by identifying a predominant deposition of IgA in the immunofluorescence study of the post-transplant graft biopsy in patients with pre-transplant IgAN diagnosis. The IgAN features were described according to the presence of mesangial hypercellularity, endocapillary hypercellularity, segmental glomerulosclerosis, tubular atrophy, and crescents on light microscopy. The cell-mediated rejection cases were treated with steroid pulse therapy or anti-thymocyte globulin. The antibody-mediated rejection treatment consisted of 5-7 sessions of plasmapheresis associated with 2g/kg intravenous immunoglobulin (IVIG).

Statistical analysis: Continuous variables are reported as mean ± standard deviation or as median and range and/or percentages. Continuous variables were compared among the groups using the Kruskal-Wallis test, whereas categorical variables were compared using Pearson χ^2^ tests. Values of p < 0.05 were considered statistically significant. Data were analyzed with the GraphPad Prism 7.0c™ for Mac (La Jolla CA, USA).

## Results

Of 2,870 kidney transplantations performed between 1999 and 2019, 53 fulfilled the inclusion criteria and were included in the study. The majority was male, white, eutrophic, with a mean age of 27 ± 9 years at IgAN diagnosis [Table 1]. Thirty-six (67.9%) patients presented systemic arterial hypertension, 28 presented proteinuria (52.8%), and 17 microscopic hematuria (50.9%). All patients had negative serology for human immunodeficiency virus (HIV), hepatitis B virus (HBV), or hepatitis C virus (HCV) infections. The description of native kidney biopsies was available for 35 cases (66.0%). Biopsies presented 50% of glomeruli with mesangial hypercellularity (M1, n = 15, 42.8%) and segmental glomerulosclerosis (S1, n = 18, 51.4%), classified according to the Oxford MEST-C criteria [Table 2]. Fourteen kidney biopsy samples (40.0%) presented tubular atrophy or interstitial fibrosis in more than one-quarter of the cortical area. Cellular or fibrocellular crescents were present in eight biopsy specimens (22.8%). In electron microscopy analysis, electron-dense deposits were observed in 11 (31.4%) cases, being eight (36.4%) in the mesangial area and three (13.6%) in the subendothelial area.

**Table 1.** Characteristics of the kidney transplant recipients with pre-transplant biopsy-proven iga nephropathy according to the post-transplant igan recurrence

	Total	Non-IgAN recurrence	IgAN recurrence	p
Total, n (%)	53	49 (92.4)	4 (7.5)	
***General***				
Male, n (%)	28 (52.8)	26 (53.0)	2 (50.0)	1.00
Ethnicity, n (%)				
White	42 (79.2)	38 (77.5)	4 (100.0)	
Brown	8 (15.1)	8 (16.4)	0 (0.0)	
Black	2 (3.8)	2 (4.0)	0 (0.0)	
Yellow	1 (1.9)	1 (2.0)	0 (0.0)
***IgA nephropathy diagnosis***				
Age at diagnosis (years)	27.0 ± 9.0	28.4 ± 9.1	18.9 ± 3.6	0.04
Body mass index (kg/m^2^)	26.4 ± 6.0	26.9 ± 5.7	20.6 ± 3.5	0.03
Systemic hypertension, n (%)	36 (67.9)	34 (69.4)	2 (50.0)	0.58
Edema, n (%)	12 (22.6)	10 (20.4)	2 (50.0)	0.22
Proteinuria, n (%)	28 (52.8)	26 (53.0)	2 (50.0)	1.00
Nephrotic syndrome, n (%)	8 (15.1)	7 (67.3)	1 (25.0)	0.49
Microscopic hematuria, n (%)	27 (50.9)	26 (53.0)	1 (25.0)	0.35
Macroscopic hematuria, n (%)	8 (15.1)	8 (16.3)	0 (0.0)	
C3 reduction	1 (1.9)	1 (2.0)	0 (0.0)	
C4 reduction	0 (0.0)	0 (0.0)	0 (0.0)	
Detectable antinuclear factor	0 (0.0)	0 (0.0)	0 (0.0)	
Family history of glomerulopathy	0 (0.0)	0 (0.0)	0 (0.0)	
***Kidney transplantation***				
Age at transplant	37.7 ± 11.2	38.8 ± 11.6	22.5 ± 4.7	<0.01
Previous kidney transplantation, n (%)	3 (5.7)	2 (4.1)	1 (25.0)	0.21
Deceased donor, n (%)	38 (71.7)	36 (73.4)	2 (50.0)	0.20
HLA A,B,DR mismatches	2.7 ± 1.6	3.0 ± 1.8	4.2 ± 0.9	0.19
Cold ischemia time (hours)	18.4 ± 7.1	18.1 ± 7.2	22.5 ± 11.9	0.27
Induction therapy, n (%)	32 (60.4)	32 (27.1)	0 (0.0)	
Basiliximab	12 (37.5)	12 (24.5)	0 (0.0)	
Thymoglobulin 3 mg/kg	7 (21.9)	7 (14.3)	0 (0.0)	
Thymoglobulin 4.5 mg/kg	8 (25.0)	8 (16.3)	0 (0.0)	
Thymoglobulin 6 mg/kg	5 (15.6)	5 (10.2)	0 (0.0)	
Delayed graft function, n (%)	19 (35.8)	3 (6.1)	0 (0.0)	

HIV: human immunodeficiency virus; HBV: hepatitis B virus; HCV: hepatitis C virus; HLA: human leukocyte antigen.

**Table 2.** Histologic features of native kidney biopsies of patients with pretransplant iga nephropathy according to igan recurrence after transplantation

	General	Non-IgAN recurrence	IgAN recurrence	p
Total, n (%)	35 (66.0)	31 (88.6)	4 (11.4)	
***Oxford classification***				
M1	15 (42.8)	13 (41.9)	2 (50.0)	1.00
E1	3 (8.6)	2 (6.4)	1 (25.0)	0.31
S1	18 (51.4)	16 (51.6)	2 (50.0)	1.00
T1	8 (14.3)	6 (19.3)	2 (50.0)	0.22
T2	6 (17.1)	6 (19.3)	0 (0.0)	
C1	6 (17.1)	5 (16.1)	1 (25.0)	0.55
C2	2 (5.7)	2 (6.4)	0 (0.0)	
***Electron-dense deposits***				
Mesangial, n (%)	8 (36.4)	7 (22.6)	1 (25.0)	1.00
Subendothelial, n (%)	3 (13.6)	3 (9.7)	0 (0.0)
Subepithelial, n (%)	0 (0.0)	0 (0.0)	0 (0.0)	

M: mesangial hypercellularity; E: endocapillary hypercellularity; S: segmental glomerulosclerosis; T: tubular atrophy and interstitial fibrosis; C: cellular and fibrocellular crescents.

The mean age at transplantation was 37.7 ± 11.2 years, about ten years older than at IgAN diagnosis, and the majority of patients received a kidney from a deceased donor (n = 38, 71.7%). In this series, three patients had previous kidney transplantation, one with graft loss due to chronic graft dysfunction and the others because of arterial thrombosis. Thirty-two patients (60.4%) received immunosuppressive induction therapy, mainly thymoglobulin (n = 20, 62.5%), associated to a routine methylprednisolone injection. During post-transplant follow-up, renal graft biopsy was performed in 20 patients (57.1%) mainly because of renal dysfunction (n = 18, 90%). Of these cases, four presented proteinuria (22.2%) and one hematuria (5.5%). One graft biopsy was indicated due to isolated proteinuria and there was no case of graft biopsy performed due to isolated hematuria. Acute rejection was diagnosed in 12 patients (22.6%), being 11 classified as cell-mediated. The cell-mediated rejection cases occurred 12.3 ± 30.2 months post-transplant, without a temporal relationship with IgAN diagnosis. Only one patient presented histologic changes compatible with chronic rejection.

Four white patients (7.5%) with a mean age of 22 ± 4.76 years presented histologic features of IgAN recurrence in a period ranging from 6 to 122 months after transplant. The IgAN recurrence group was younger at IgAN diagnosis in native kidney (p = 0.04) and at kidney transplant (p = 0.01) [Table 1]. These patients were eutrophic (BMI of 20.6 ± 3.5 kg/m^2^), while the non-recurrent patients were classified as overweight, with a significantly higher mean BMI (p = 0.03). In this series, all the IgAN recurrence patients did not receive immunosuppressive induction therapy at transplant, and the immunosuppressive maintenance regimen included a calcineurin inhibitor, an antiproliferative drug, and steroids. The mean dose of prednisone in the IgAN recurrence group was 2.5 mg daily, while in the group without IgAN recurrence was 5 mg daily (p = 0.01). The native kidney biopsy registries of IgAN recurrence group showed mesangial hypercellularity in more than 50% of glomeruli (M1), segmental glomerulosclerosis (S1), and tubular atrophy and interstitial fibrosis in 26% to 50% of the cortical area (T1) [Table 2]. One case presented endocapillar hypercellularity (E1) and cellular or fibrocellular crescents in up to a quarter of glomeruli (C1). Electron-dense deposits in the mesangial area were detected in one case (50.0%). There was no significant difference in Oxford classification of native kidney biopsies in the IgAN recurrence group compared to the group without IgAN recurrence.

Long term follow-up of renal transplants from patients with native IgAN showed 17 (32%) graft failures 41.3+ 38.1 months after transplant, being four (23.5%) due to IgAN recurrence, six (35.3%) secondary to chronic graft dysfunction without recurrence, and two (11.8%) due to acute rejection. Five patients died with a functioning graft (29.4%).

## Discussion

The general characteristics of the patients with biopsy-proven IgAN in this study are similar to those of previous studies, with patients younger than other causes of chronic kidney disease (CKD)^8^. Most patients had arterial hypertension and proteinuria at IgAN diagnosis in native kidneys, which are considered risk factors for the progression to chronic kidney disease^9^. Morphological analysis of native kidney biopsies showed mesangial hypercellularity in 42.8% of patients, and prevalence of segmental glomerulosclerosis higher than 50%, evidencing its impact on the progression to kidney failure. Compared with other causes of CKD, patients with IgAN have better access to kidney transplantation, since they are younger, have significantly fewer comorbidities, and have a shorter waiting-list time^2^.

The post-transplant IgAN recurrence is relatively common, with recurrence rates ranging from 8% to 53%^2,9^, differing according to the biopsy indication and length of post-transplant follow-up^2^. In this series, the IgAN recurrence rate was 7.5%, possibly due to the lack of protocol graft biopsies, limiting the diagnosis of recurrence to cases of graft dysfunction or proteinuria. However, protocol biopsies performed exclusively to detect IgAN recurrence are not recommended, as no treatment has been shown to halt or slow the progression of clinical disease^2^. In this series, the age at native IgAN diagnosis and transplantation were significantly lower in the IgAN recurrence group, similar to other studies^10^. The determinants for IgAN recurrence are poorly understood, but several characteristics have been identified as possible factors, such as younger age at transplantation, rapid progression of native IgAN, level of proteinuria, and donor characteristics^2^. Several studies have shown the association between glomerular C4d and progression of IgAN^11^. In this series, however, the IgAN recurrence biopsies were performed before establishing the C4d research protocol; therefore, we did not have this information. IgAN recurrence was associated with young age and long-term posttransplant follow-up in this series, concordant to previous studies^9^. The BMI was significantly lower in the IgAN recurrence group. Although higher body mass index (BMI) was associated with IgAN progression^12^, little is known about its influence on IgAN recurrence.

The heterogeneity in immunosuppressive protocols seems to be also associated with IgAN recurrence, such as immunosuppressive protocols free of induction therapy, steroid-avoidance, or early steroid-withdrawal^3^. A retrospective study of Berthoux et al.^13^ showed a 9% IgAN recurrence rate after anti-thymocyte globulin therapy, while the group without induction presented a 41% recurrence rate. Although the IgAN recurrence group did not receive induction immunosuppression and received a significantly lower maintenance dose of steroids than the group without IgAN recurrence in this series, the IgAN recurrence rate was lower than that study. We hypothesized that this could be related to all these cases taking a immunosuppressive maintenance regimen with a calcineurin inhibitor, antiproliferative drugs, and steroids. However, the protective effect of immunosuppressive regimen in IgAN recurrence patients remains controversial since it was not verified in some studies^10^.

The optimal treatment for IgAN recurrence remains unknown^3^. In this series, the patients with IgAN recurrence did not receive additional specific treatment. Previous studies showed that treatment of IgAN recurrence with ACEi, BRA, pulse steroids, or intravenous cyclophosphamide had no impact on the outcomes^10^. In this series, all patients with high scores in the Oxford classification were associated with poor prognosis^14^. Although the Oxford classification has been developed in native kidneys, it has also been successfully applied for IgAN recurrence in transplant kidneys^2^. The patients with IgAN recurrence presented high scores in the native kidney biopsies, and mesangial hypercellularity was detected in all IgAN recurrence graft biopsies. A multicenter, international, retrospective study by Uffing et al.^10^ showed that IgAN recurrence was associated with a 3.7-fold higher risk of graft loss. A study by Nijim et al.^15^ showed a mean graft survival of 6.5 ± 5.1 years. Graft survival time after IgAN recurrence diagnosis was similar in this series, with a median survival of 68.5 months. The limitations of this study are its single-center and retrospective design. Besides this, the lack of protocol biopsies impaired the accuracy of the true prevalence of IgAN recurrence in the center, which was probably underestimated in the present study.

## Conclusions

IgAN is the most common glomerular disease with relatively common recurrences, varying widely according to biopsy indication and length of post-transplant follow-up. In this series, IgAN recurrence was associated with younger age and long-term post-transplant follow-up. According to the Oxford classification, there was a high prevalence of mesangial hypercellularity and segmental glomerulosclerosis on native kidney biopsies, and mesangial hypercellularity occurred in all IgAN recurrence graft biopsies. The progression to graft loss was 100% in the IgAN recurrence group, and lower doses of maintenance steroids were associated with a worse prognosis.
